# Could immunotherapy be a hope for addiction treatment?

**DOI:** 10.1016/j.clinsp.2024.100347

**Published:** 2024-04-06

**Authors:** Maria Carolina Machado da Silva, Luiz Philipe de Souza Ferreira, Amanda Della Giustina

**Affiliations:** a Neuropharmacology Laboratory, Department of Pharmacology, Universidade Federal de Minas Gerais, Minas Gerais, BH, Brazil; b Department of Morphology and Genetics, Structural and Functional Biology Graduate Program, Escola Paulista de Medicina, Universidade Federal de São Paulo, São Paulo, SP, Brazil; c Sprott Centre for Stem Cell Research, Ottawa Hospital Research Institute, Ottawa, ON, Canada

Substance use disorder (SUD) or addiction is defined as a chronic illness in which there is physical and psychological dependence on psychoactive substances. It is characterized by compulsive drug-seeking behavior, lack of self-control during use, and negative physiological and psychological changes (e.g., irritability, anxiety, and dysphoria) in the absence of the substance.^1^^,^^2^

According to the World Drug Report 2023, it is estimated that 296 million people used psychoactive substances in 2021 and approximately 40 million have developed substance use disorder.^3^ Moreover, the number of deaths resulting from psychoactive substance misuse reached around 500.000 in 2019. Finally, even if it does not lead to death, in 2021, the use of drugs generated a “loss of healthy life” of approximately 32 million years.^3^

Despite the deleterious effects of drug use being widely known, the prevalence of people who use drugs remains high, which is intrinsically related to the mechanism of action of drugs of abuse. According to DSM-5, psychoactive substances encompass ten distinct classes of drugs: stimulants, caffeine, alcohol, tobacco, marihuana, opioids, anxiolytics, sedatives and hypnotics, inhalants, hallucinogens, and other unknown substances.^1^ Despite being divided into different categories and presenting various neuropharmacological properties, the psychoactive substances act directly on the reward system,^4^ which is formed mainly by the Ventral Tegmental Area (VTA), the Nucleus Accumbens (NAc) and the Prefrontal Cortex (PFC),^5^ promoting an imbalance in the levels of neurotransmitters in the mesocorticolimbic dopaminergic and in the corticolimbic glutamatergic pathways.^2^^,^^6^^,^^7^ Consequently, psychoactive substances reorganize and promote plastic changes in these circuits of the Central Nervous System (CNS), “hijacking” neural adaptive motivational mechanisms,^8^, ^9^, ^10^ and leading to the dysfunctional pattern of behavior that characterizes drug addiction.^1^

Importantly, these neuroplastic changes that occur after drug exposure are so forceful that, even after extensive periods of abstinence, the drug's reinforcing effects are still present, leading to high rates of relapse and being a challenge to treat.^11^ In fact, there are very few pharmacological options to treat alcohol, nicotine, and opioid use disorder, and no pharmacotherapies for substances such as psychostimulants (cocaine, methamphetamines) and marijuana/synthetic cannabinoids. In addition, the treatment adherence rate is extremely low, with a dropout prevalence of almost 90%.^12^^,^^13^ Thus, there is an urgent clinical need for extensive research to develop new molecular targets and pharmacological options.

Although alterations in the dopaminergic and glutamatergic systems are considered key in the neurobiological changes that regulate motivated behavior, it is known that psychoactive substances can also alter other molecular pathways, including immunologic signaling.^14^, ^15^, ^16^ For example, alcohol, opioids, and psychostimulants can alter microglia morphology, microglial activation markers, and cytokines levels in preclinical and clinical studies.^17^, ^18^, ^19^ During the years, glial cells were described as supportive cells for neurons. However, a growing body of evidence now indicates that both microglia and astrocytes can regulate neuronal circuits, actively participating in processes such as neurogenesis, neurotransmitter release, modulation of synaptic morphology, and neuronal connectivity.^20^^,^^21^ In this sense, the interaction between psychoactive substances and the microglia and astrocytes could, directly or indirectly, contribute to the alterations in brain function and the behavioral changes that occur in substance use disorder. For example, a study using the radioligand [11C](*R*)-PK11195 showed increased microglial activity in the midbrain, striatum, thalamus, and the orbitofrontal and insular cortex from abstinent METH abusers, which was negatively correlated with the duration of abstinence.^22^ In addition, increased IL-1β production due to polymorphisms in *IL1B* gene is associated with an increased risk of opioid and alcohol dependence in humans,^23^ while IL-6 is associated with METH-induced mesocorticolimbic functional connectivity.^24^ Once these cytokines can be produced by the glial cells and are important for CNS neuroplasticity, could the inhibition of drug-induced neuroinflammation be a pharmacological approach against addiction?

Although the literature is still scarce, some studies have shown the beneficial effects of immunomodulators in substance use disorder treatment. Inhibition of microglial activation by minocycline, a tetracycline antibiotic widely used as a microglial inhibitor, reverses the behavioral alterations and dopamine release induced by cocaine^25^^,^^26^ and methamphetamine^27^^,^^28^ in mice. Ibudilast, an anti-inflammatory drug, also reduces the behavioral sensitization and the self-administration of cocaine by rats^29^^,^^30^ and ethanol intake in three different rodent models of alcohol use disorder.^31^ Chronic ethanol intake and relapse are also reduced by aspirin, a non-steroidal anti-inflammatory drug, in rats.^32^ Finally, the selective COX-2 inhibitors – valdecoxib and LM-4131 – attenuated nicotine preference,^33^ while rofecoxib and nimesulide protected against withdrawal symptoms induced by alcohol.^34^

Some clinical studies have also evaluated the potential of anti-inflammatory/immunomodulators in the SUD. Minocycline improved the psychotic symptoms of METH use disorder in a female patient. Besides, this drug also reduced some of the subjective reinforcing effects of d-amphetamine,^35^ oxycodone^36^ and the craving for cigarettes.^37^ The anti-inflammatory ibudilast decreased some reward-related as well as peripheral inflammatory markers in METH-dependent volunteers.^38^^,^^39^ Also, it reduced cocaine and heroin craving in human volunteers diagnosed with opioid dependence,^30^^,^^40^^,^^41^ decreased withdrawal symptoms in heroin-dependent patients,^42^ decreased the positive subjective and reinforcing effects in opioid-dependent.^40^ Ibudilast also decreased craving for alcohol in a small, randomized, placebo-controlled, and human laboratory trial. The authors suggest that this effect may be due to the anti-inflammatory properties of this drug.^43^^,^^44^

In general, minocycline and ibudilast are inexpensive drugs that are also well-tolerated and induce only moderate side effects. For example, in a methamphetamine clinical trial, there is no difference in the rate of adverse effects between Ibudilast placebo and groups.^45^ Despite this promising data and the potential benefits of these compounds, more studies are necessary for a better comprehension of the clinical use of immunomodulators in substance use disorder.

In summary, alterations in neuroimmune signaling are emerging as an important contributing factor in the neurobiology of substance use disorder. The understanding of how glial and neuroinflammatory responses modulate the development and maintenance of this disease could provide novel insights and contribute to the development of new pharmacological targets.

## Declaration of competing interest

The authors declare no conflicts of interest.
